# Functionalization of an Antisense Small RNA

**DOI:** 10.1016/j.jmb.2015.12.022

**Published:** 2016-02-27

**Authors:** Guillermo Rodrigo, Satya Prakash, Teresa Cordero, Manish Kushwaha, Alfonso Jaramillo

**Affiliations:** 1 Instituto de Biología Molecular y Celular de Plantas, Consejo Superior de Investigaciones Científicas, Universidad Politécnica de Valencia, 46022 Valencia, Spain; 2 School of Life Sciences, University of Warwick, Coventry CV4 7AL, United Kingdom; 3 Institute of Systems and Synthetic Biology, Centre National de la Recherche Scientifique, Université d'Evry val d'Essonne, 91000 Évry, France

**Keywords:** biotechnology, computational design, information encoding, regulatory RNA, synthetic biology, sRNA, small RNA, 5′ UTR, 5′ untranslated region, RBS, ribosomal binding site, GFP, green fluorescent protein, aTc, anhydrotetracycline, mRNA, messenger RNA, IPTG, isopropyl-β-D-1-thiogalactopyranoside

## Abstract

In order to explore the possibility of adding new functions to preexisting genes, we considered a framework of riboregulation. We created a new riboregulator consisting of the reverse complement of a known riboregulator. Using computational design, we engineered a *cis*-repressing 5′ untranslated region that can be activated by this new riboregulator. As a result, both RNAs can orthogonally *trans*-activate translation of their cognate, independent targets. The two riboregulators can also repress each other by antisense interaction, although not symmetrically. Our work highlights that antisense small RNAs can work as regulatory agents beyond the antisense paradigm and that, hence, they could be interfaced with other circuits used in synthetic biology.

## Highlights

• We have engineered a riboregulator as the negative-sense strand of another riboregulator. This new RNA molecule performs the cellular function of titration of a functional molecule or *trans*-activation of gene expression.
• We have followed a computational design approach with energetic and structural criteria to obtain the nucleotide sequence of a 5′ untranslated region responding to the new riboregulator.
• We have engineered different regulatory circuits with *trans*-activating and anti-*trans*-activating small RNAs and characterized them at the population and single-cell levels.

## Graphical abstract

**Figure:**
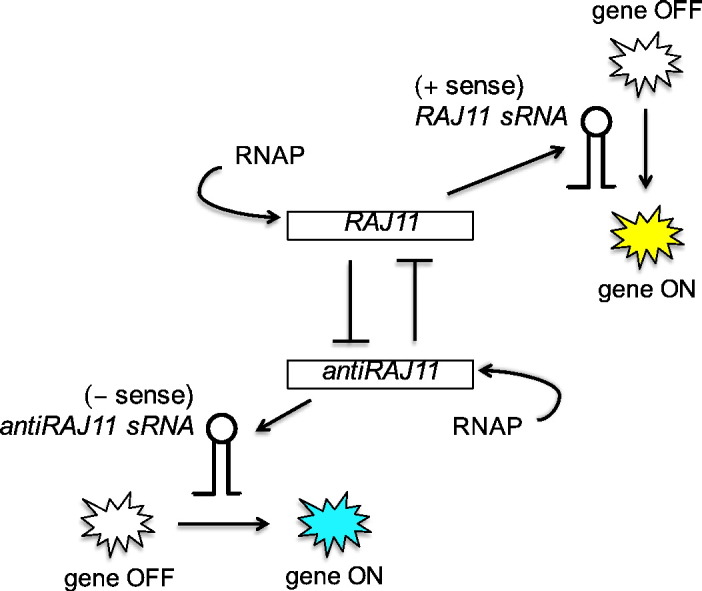

Genetic material is, paraphrasing the words of Alphonse de Lamartine brought to this context, *limited in its nature but infinite in its desires*. Four combinatorial bases offer the possibility of encoding vast information [1], and even the same sequence can provide, in some cases, multiple functions [2]. In this work, we investigate the possibility of adding a new function to an existing gene. To this end, we focused on DNA sequences that produce transcripts able to perform a cellular function without being translated (i.e., regulatory RNAs) [3]. In particular, we envisioned a scenario wherein an antisense small RNA (sRNA) can *trans*-sequester a functional sRNA that targets an existing gene. This regulatory mechanism has already been described in prokaryotes [4], [5] and eukaryotes [6], [7], and this work will show that an antisense sRNA may additionally acquire the ability to directly control gene expression. We present a synthetic case in *Escherichia coli*, where the two transcribed sRNAs, one the reverse complement of the other, can independently activate gene expression (Fig. 1a). We used a *de novo* sequence design methodology to obtain the sequence of a new riboregulator device that we named antiRAJ11 based on our previously engineered riboregulator RAJ11 [8].

Indeed, there is a growing interest, and need, to use non-coding RNAs rather than proteins in synthetic biology [9]. The reasons are their greater *ab initio* predictability of folding and interaction ability (i.e., function) [8], [10], [11], [12], [13], their broad repertoire of regulatory mechanisms [14], [15], [16], [17], [18], [19], and the faster action compared to regular transcription factors [5], [20], [21]. Here, we focused on bacterial riboregulators (sRNAs), having the ability to induce a conformational change in a specific 5′ untranslated region (5′ UTR) of a messenger RNA (mRNA) to modulate gene expression [8], [14]. The secondary structure of the 5′ UTR allows base pairing of ribosomal binding site (RBS), which then becomes inactivated to recruit the 16S ribosomal RNA. Binding of the sRNA to the 5′ UTR of the mRNA results in the release of the RBS (*via* a conformational change), and the RBS is then converted into a fully functional motif.

The sRNA antiRAJ11 was designed as the reverse complement of the sRNA RAJ11 (without including transcription terminators). Using RiboMaker [22] as computational method and following defined energetic and structural criteria (Fig. S1; see details in supplemental information), we designed a 5′ UTR sequence that is able to *cis*-repress the RBS (thus blocking the translation) and to be *trans*-activated by the new riboregulator antiRAJ11 (Fig. 1a). The RNA sequences are shown in Fig. S2 and the plasmid maps are in Figs. S3, S4, and S5. The heuristic algorithm performs multiple cycles of random mutations and selection, and each run produces a different sequence. Hence, we ran RiboMaker multiple times and selected the best sequence according to the desired function (see details in supplemental information). Note that it is not possible to construct the 5′ UTR antiRAJ11 as the reverse complement of the 5′ UTR RAJ11 because it would lack a suitable RBS sequence. Moreover, riboregulatory systems require a precise secondary structure, followed by toehold formation, which is not maintained after a reverse complementation operation due to the existence of GU wobble pairs.

We used inducible promoters [23] to dynamically control the expression of our system with isopropyl-β-d-1-thiogalactopyranoside (IPTG) and anhydrotetracycline (aTc) (Fig. 1b). As reporter, we used a green fluorescent protein (GFP). The design of the full expression cassette including the promoters and terminators of the sRNA and mRNA was performed according to a previously described protocol [24]. The characterization of the system *in vivo* at the population level revealed a high dynamic range and a 64-fold activation with aTc (Fig. 1b). Moreover, the single-cell analysis also revealed that the whole population shifted to the ON state upon induction with IPTG and aTc (Fig. S6), while the population was maintained in the OFF state upon induction with only one chemical.

We then tested the ability of the riboregulator RAJ11 to inhibit the action of the riboregulator antiRAJ11, as they hybridize perfectly with each other. When co-expressing both riboregulators, we observed a remarkable decrease in GFP expression (Fig. 1c). We also tested the ability of the riboregulator antiRAJ11 to inhibit the action of the riboregulator RAJ11, obtaining a reduction in GFP expression but less substantial than in the previous case (Fig. S7). We decided to perform a Boolean assay (i.e., with/without antisense sRNA) because, in our constructions, the sRNAs (RAJ11 and antiRAJ11) are expressed from the same promoter. Using *in vitro* translation (see details in supplemental information) wherein the complementary DNAs (cDNAs) corresponding to the RNA species were first transcribed *in vitro*, we also proved the dynamic behavior of the new system antiRAJ11 and the inhibitory role of sRNA RAJ11 (Fig. 1d, see also Fig. S8). Consequently, we observed that the appropriate activation of the target genes, i.e., genes controlled either by the system RAJ11 or by antiRAJ11, could require non-simultaneous expression regimes of the riboregulators.

In addition, we studied to what extent one riboregulator can affect the targets of the other (i.e., if they are orthogonal). For this, we measured the change in GFP expression from the mRNA controlled by the non-cognate 5′ UTR in the presence or absence of the sRNA (i.e., crossed systems). We found that the riboregulator antiRAJ11 has no significant impact on the GFP controlled by the 5′ UTR RAJ11 (Fig. S9), and the same applies for the riboregulator RAJ11 on the GFP controlled by the 5′ UTR antiRAJ11 (Fig. S10). We also found that, in both cases, the 5′ UTRs are very efficient at repressing translation. Simulations with an RNA physicochemical model [10] revealed no significant free energy of hybridization between the non-cognate sRNAs and mRNAs, supporting the orthogonal behavior. Finally, to gain mechanistic insights, we performed a native polyacrylamide gel electrophoresis (PAGE; see details in supplemental information), [25] where the cDNAs were again first transcribed *in vitro*. We mixed two species per lane, by adjusting the amount of each RNA. The gel revealed the intermolecular interactions between the sRNA and 5′ UTR of systems RAJ11 and antiRAJ11, as well as the interaction between the two riboregulators while no interaction was detected for the non-cognate pairs.

In conclusion, we have demonstrated that it is possible to use an antisense sRNA as a new regulatory agent in the cell. This was accomplished by designing an appropriate 5′ UTR. These results largely indicate the development of sophisticated RNA-only circuits where sRNAs interact with each other to form arbitrary regulatory architectures. We also hypothesize that antisense transcripts [4] could be exploited in synthetic biology as overlapping reading frames of transcription (i.e., ambisense) for engineering bi-functional systems with minimal genetic material, as well as non-linear behavior with RNA, as convergent transcription has been shown to confer a bi-stability [26].

## Figures and Tables

**Fig. 1:**
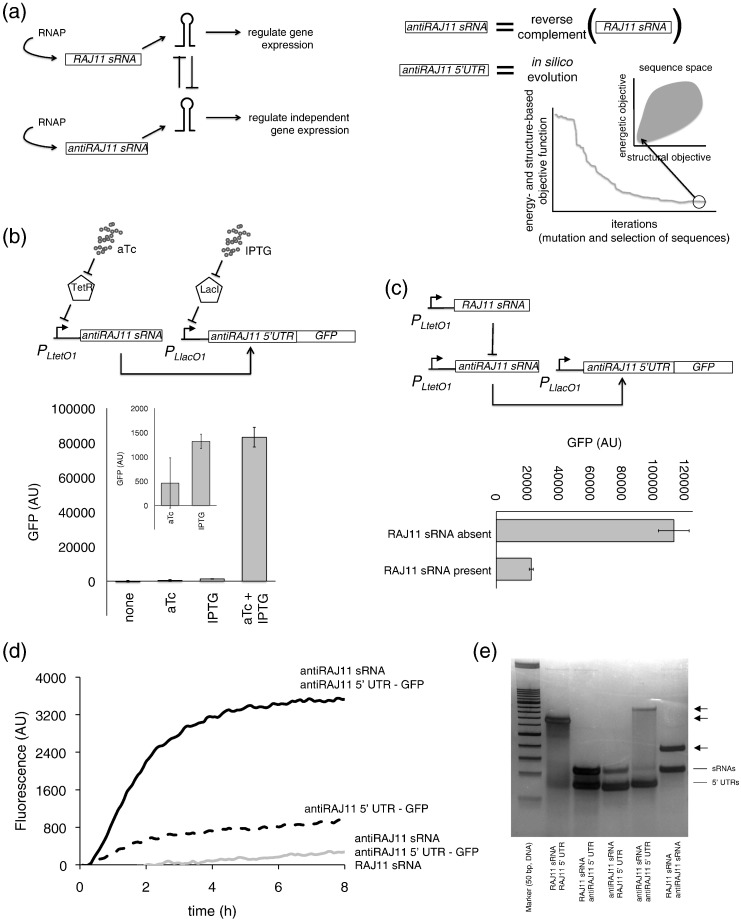
Design and characterization of a riboregulator as the negative-sense strand of the riboregulator RAJ11. (a) On the left, scheme of the circuit of system antiRAJ11. Here, antiRAJ11 sRNA is the negative-sense strand of our previous RAJ11 sRNA. On the right, illustration of the computational design of the 5′ UTR regulated by an antiRAJ11 sRNA. Note that the two sRNAs are functional. (b) Characterization result of system antiRAJ11 *in vivo* with the appropriate inducers (aTc and IPTG). Assays performed in MGZ1 cells. (c) Scheme of the regulatory circuit and characterization result of RAJ11 sRNA inhibition of the action of antiRAJ11 sRNA. Here, RAJ11 sRNA is absent (only plasmid pMIR03) or present (introduced with a different plasmid; pMIR03 + pMIR04). Assays performed in JS006 cells. (d) Characterization result of system antiRAJ11 by *in vitro* translation. (e) Molecular characterization of the RNA–RNA interactions by native polyacrylamide gel electrophoresis (PAGE). Arrows indicate intermolecular complexes. (d and e) RNAs were pre-transcribed with T7 polymerase and purified.
