# Supplementary material for: Functionalization of an Antisense Small RNA
Source: J Mol Biol. 2016 Feb 27;428(5Part B):889–92. doi: 10.1016/j.jmb.2015.12.022 (PMC4819895; doi:10.1016/j.jmb.2015.12.022)
Supplement: Supplementary file 1 — Supplementary material. [file mmc1.docx]

**SUPPLEMENTAL INFORMATION**

**Functionalization of an antisense small RNA**

Guillermo Rodrigo1,#, Satya Prakash2,#, Teresa Cordero1, Manish Kushwaha2,

and Alfonso Jaramillo2,3,*

*1 Instituto de Biología Molecular y Celular de Plantas, CSIC – Universidad Politécnica de*

*Valencia, 46022 Valencia, Spain.*

*2 School of Life Sciences, University of Warwick, Coventry, CV4 7AL, United Kingdom.*

*3 Institute of Systems and Synthetic Biology, CNRS – Université d’Evry val d’Essonne, 91000*

*Évry, France.*

*# Equal contribution.*

**Materials and Methods**

**Computational sequence design**

The sequence of the RAJ11 sRNA was used as a template ^1^. The sequence of the antiRAJ11 sRNA was obtained as the negative-sense strand (reverse complement) of the RAJ11 sRNA (excluding the transcription terminator). In our original designs, the RAJ11 sRNA has the B0015 terminator, and the antiRAJ11 sRNA the T500 terminator (see ref. [2] for details). In order to design the sequence of the 5' UTR regulated by the antiRAJ11 sRNA, we used the web-based software RiboMaker ^3^ to automatically design regulatory RNAs that exploit conformational changes to control gene expression. It uses a Monte Carlo simulated annealing optimization algorithm ^4^ and the ViennaRNA package ^5^ to compute the free energies and secondary structures of different species in the system. Here, the system comprised three RNA molecules: the small RNA (sRNA), the messenger RNA (mRNA, with the 5' UTR), and the interacting complex. To implement this algorithm, we constructed a physicochemical model based on free energies and RNA structures that included the energies of activation and hybridization corresponding to the interaction between the antiRAJ11 sRNA and its cognate 5' UTR. The model also accounted for the degree of repression and release of the ribosomal binding site (RBS) within the 5' UTR intramolecular and intermolecular structures. Successive rounds of random mutations over the 5' UTR sequence were applied and selected with an energy-based objective function (Fig. S1). Designed sequences are shown in Fig. S2.

Ribomaker was run several times to design the 5' UTR responding to the antiRAJ11 sRNA. The heuristic algorithm performs multiple cycles of random mutations and selection, and each run produces a different sequence. The objective function accounts, on one side, on the *cis*-repression of the 5' UTR, and, on the other, on the interaction ability with the sRNA. Ribomaker outputs the values of effective free energies (to be minimized) corresponding to all partial objectives; and the global objective function is a sum of these free energies. From all sequences designed, we selected the one with minimal value of objective function.

**Plasmid construction**

The plasmids were constructed with standard cloning methods ^6^. For ease of cloning, each system was flanked by EcoRI and SpeI sites, allowing BioBrick compatibility. The sRNA systems (from the sRNA – in reverse orientation– to the 5' UTR) of pMIR01, pMIR02, and pMIR03 were synthesized by integrated DNA technologies (IDT) as gBlocks, digested with EcoRI and SpeI and ligated into the final vector (pSTC1 ^1^), and then transformed into TOP10 cells (Invitrogen). In addition, pMIR04 was constructed by removing the reporter gene from our pSTC0 ^1^, digested with PstI and NsiI, and self-ligated, while pMIR06 was constructed by removing the reporter gene from pMIR03 and cloned with EcoRI and NsiI into pSTC0. Finally, pMIR05 was constructed by cloning the RAJ11 cassette with EcoRI and PstI into pSTC1. Plasmids were digested with appropriate restriction enzymes, purified by gel extraction, and dephosphorylated to prevent self-ligation. Enzymes were obtained from Thermo Scientific and DNA extraction kits from Qiagen.

The different sRNA systems were cloned either in a pSTC1-based plasmid that contained a pSC101 replication origin with a mutation in the origin of replication (ori) giving a high copy number, and a kanamycin resistance marker (plasmids pMIR01, pMIR03, and pMIR05) ^7^, or in a pSTC0-based plasmid that contained a pMB1 replication origin (giving a high copy number) and an ampicillin resistance marker (plasmids pMIR02, pMIR04, and pMIR06) ^1^. In order to construct this latter plasmid, we removed the kanamycin resistance marker present in the native pSTC0 by digesting it with XhoI.

In our designs, the PLlacO1 and PLtetO1 promoters ^8^ control the expression of the mRNA and sRNA, respectively, in the engineered antiRAJ11 system. In other constructs, the mRNA was constitutively expressed from J23119 promoter. We used a superfolder green fluorescent protein (sfGFP) ^9^ with an *ssr*A degradation tag ^10^ as reporter for fast turnover. Plasmids used in this study are shown in Fig. S3, S4, and S5.

**Cell culture and reagents**

*Escherichia coli* strain TOP10 was used for plasmid construction purposes as described in the manual ^6^. Characterization experiments were performed in *E. coli* MGZ1 cells (strain K-12 MG1655 with Z1 cassette, *lacI*+ *tetR*+) for control over the PLlacO1 and PLtetO1 promoters ^8^ or in *E. coli* JS006 cells (strain K-12 MG1655 with Δ*lacI* Δ*araC*) for constitutive expression ^11^. Cells were grown aerobically in Luria–Bertani (LB) medium or M9 minimal medium, prepared with M9 salts (Sigma-Aldrich), glycerol (0.8%, vol/vol) as the only carbon source, CaCl_2_ (100 µM), MgSO_4_ (2 mM), and FeSO_4_ (100 µM). The antibiotic concentrations were 50 µg/mL kanamycin, 100 µg/mL ampicillin, and 100 µg/mL spectinomycin. IPTG of 1 mM (Thermo Scientific) was used for full activation of the PLlacO1 promoter when needed, and 100 ng/mL aTc (Sigma-Aldrich) was used for full activation of the PLtetO1 promoter.

**Fluorescence quantification**

Single colonies were picked and inoculated into 2 mL of LB medium and grown overnight (16 h) at 37 °C and 200 rpm. The overnight culture was diluted to 1:200 in 2 mL of M9 medium with an appropriate antibiotic marker. The refreshed culture was grown at 37 °C and 200 rpm for 2 h. Then, 200 µL of culture was plated per well into a 96-well plate (Greiner). Appropriate inducers (none, aTc, IPTG, or aTc + IPTG) were introduced when needed. Characterization was done using a Tecan Infinite F500 fluorometer at 37 °C with orbital shaking by measuring absorbance (600-nm absorbance filter) and fluorescence (465/35-nm excitation filter, and 530/25-nm emission filter) every 15 min. Nine replicates (three clones and three technical replicates per clone) were assayed per sample. Experiments were also repeated on different days to ensure reproducibility.

Normalized fluorescence was obtained by subtracting the background values corresponding to M9 medium (in both fluorescence and absorbance values) and then dividing fluorescence by absorbance at OD600 ≈ 0.5. Corrected normalized fluorescence (used for representation) was then obtained by subtracting the fluorescence of plain cells (see Fig. S7, S9, and S10).

**Flow cytometry analysis**

A single colony was inoculated into 2 mL of LB medium with spectinomycin and kanamycin to maintain the Z1 cassette and plasmid (pMIR03). The overnight culture (at 37 °C and 200 rpm) was refreshed in 2 mL of LB medium with a dilution of 1:200, introducing the appropriate inducers (none, aTc, IPTG, or aTc + IPTG). The culture was grown for 3 h at 37°C and 200 rpm to reach the exponential phase. The harvested cells were washed twice and diluted (1:50) in filtered PBS (phosphate-buffered saline). All expression data were analyzed using a Becton-Dickinson FACScan flow cytometer with a 488-nm argon laser for excitation and a 530/30-nm emission filter. Data were analyzed with CytoBank (Fig. S6).

**In vitro RNA–RNA interaction**

We first constructed the complementary DNAs (cDNAs) of the different RNA species of the designed system to then perform in vitro transcription. We analyzed the systems RAJ11 and antiRAJ11. We considered the sRNAs without transcription terminators and the 5' UTR until the start codon. Amplification by polymerase chain reaction (PCR) using Phusion DNA polymerase (Thermo Scientific) was done over the template plasmids. The PCR products were cloned into the plasmid pUC18, where the restriction site Eco31I was previously removed. The resulting plasmids with inserts were selected by DNA cleavage with appropriate restriction enzymes. Sequences were also verified by sequencing.

In order to perform *in vitro* transcription, 3 µg of each pUC18-derived plasmid was digested with Eco31I and purified with silica-based columns (Zymo). We used approximately 1 µg of digested plasmid in the reaction. The reaction mixture (20 µL) contained 10 µL of plasmid, 2 µL of 10x buffer (Roche), 0.4 µL 10 mM DTT, 1 µL 10 mM NTPs (Thermo Scientific), 0.5 µL Ribolock (~40 U/µL, Thermo Scientific), 1 µL inorganic pyrophosphatase (0.1 U/µL, Thermo Scientific), 1 µL T7 RNA polymerase (50 U/µL, Epicentre), and 4.1 µL H2O. We incubated the mix for 1 h at 37 ºC and then added 20 µL of loading buffer with formamide. The samples were heated at 95 ºC for 1.5 min, cooled on ice, and subsequently separated by polyacrylamide gel electrophoresis (PAGE) in a 10% polyacrylamide gel, containing 8 M urea and 1x TBE (200 V, 2.5 h). The bands corresponding to the full-length RNAs were cut for purification. The presence of RNA was confirmed by loading a small part of the purified preparations in another polyacrylamide gel.

For the RNA–RNA interactions, we used approximately the same amount of RNA for each of the transcripts (50 ng). The buffer of the reaction was 50 mM Tris-HCl (pH 7.5), 10 mM MgCl_2_, and 20 mM NaCl. The mix (20 µL) was denatured (1.5 min at 95 ºC) and slowly cooled (15 min at room temperature). We then added 1.5 µL glycerol (87%) and 0.2 µL bromophenol blue–xylene cyanol (100x) to load the gel (15% polyacrylamide, TAE (tris, acetate, and ethylenediaminetetraacetic acid) buffer, 1-mm thick), which was run for 2 h at 75 mA at 4 ºC. The gel was stained first with ethidium bromide and then with silver.

**In vitro translation**

PCR was used to prepare DNA for *in vitro* transcription. T7 promoter was added upstream of the expression cassettes consisting of the small RNAs (RAJ11 and antiRAJ11 trans elements), or the 5' UTRs (RAJ11 and antiRAJ11 cis elements) and fluorescent gene (sfGFP). The primers and plasmid templates used are listed below:

|  | **PCR product** | **Primer (forward)** | **Primer (reverse)** | **Template DNA** | **Amplicon size** |
| --- | --- | --- | --- | --- | --- |
| 1 | RAJ11 sRNA | T7RAJ11_FW  5’-TAATACGACTCACTATA GGGAGGGTTGATTGTGTGAG-3’ | T7RAJ11_RW  5’-GCCTCGCATAAATCTGTCA CAG3’ | pMIR05  (this study) | 88 bp |
| 2 | antiRAJ11 sRNA | T7antiRAJ11_FW  5’-TAATACGACTCACTATAGG GCCTCGCATAAATCTGTCACAG-3’ | T7antiRAJ11_RW  5’-GGGAGGGTTGATTGTGTA AGTC-3’ | pMIR03  (this study) | 90 bp |
| 3 | antiRAJ11 mRNA | T7antiRAJ11GFP_FW  5’-TAATACGACTCACTATAGGGA GAGGGAGGGTTCTGTCACT TAATTATTCG-3’ | GFP_RW  5’-AGCGTAATAACTGCAGGAGT CACTAA-3’ | pMIR03  (this study) | 856 bp |

RNA was prepared by transcription using the TranscriptAid T7 High Yield Transcription Kit (Thermo Scientific #K0441) and template DNA was subsequently removed by DNase I digestion according to the manufacturer’s protocol. RNA was purified by extraction once with acid phenol:chloroform (1:1) and twice with chloroform:isoamyl alcohol (24:1). The purified RNA was used for in vitro translation using the PURExpress In Vitro Protein Synthesis Kit (New England Biolabs #E6800S). Each mRNA and sRNA was used at end concentration of 10 nM and 10 µM, respectively. The reaction volumes were scaled down to 10 µL each and prepared at 4 °C. The reactions were incubated at 37 °C, and green fluorescence was monitored using the Mx3005P qPCR System (Agilent Technologies) with filter set FAM/SYBR Green I (492 nm - 516 nm) (Fig. S8).

**Supplementary Figures**


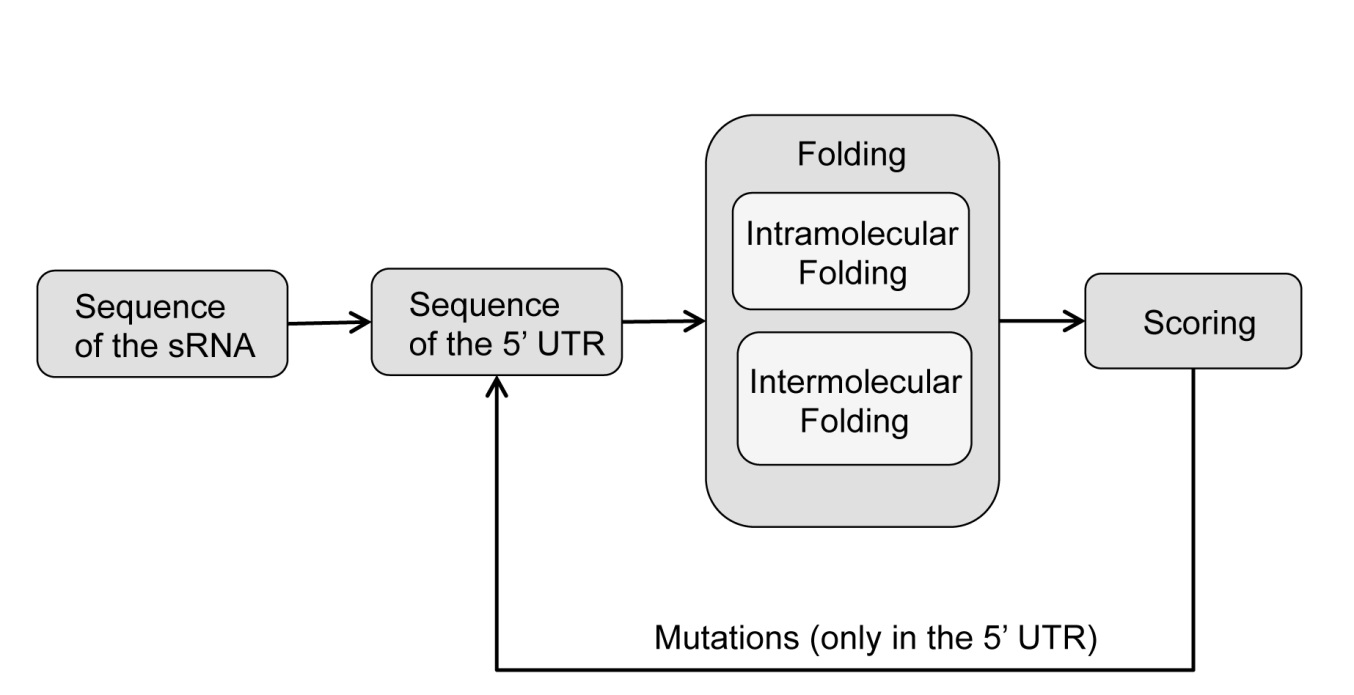


**Figure S1**: Scheme of the optimization loop to obtain a 5' UTR sequence able to interact with the antiRAJ11 sRNA, whose sequence is kept fixed.


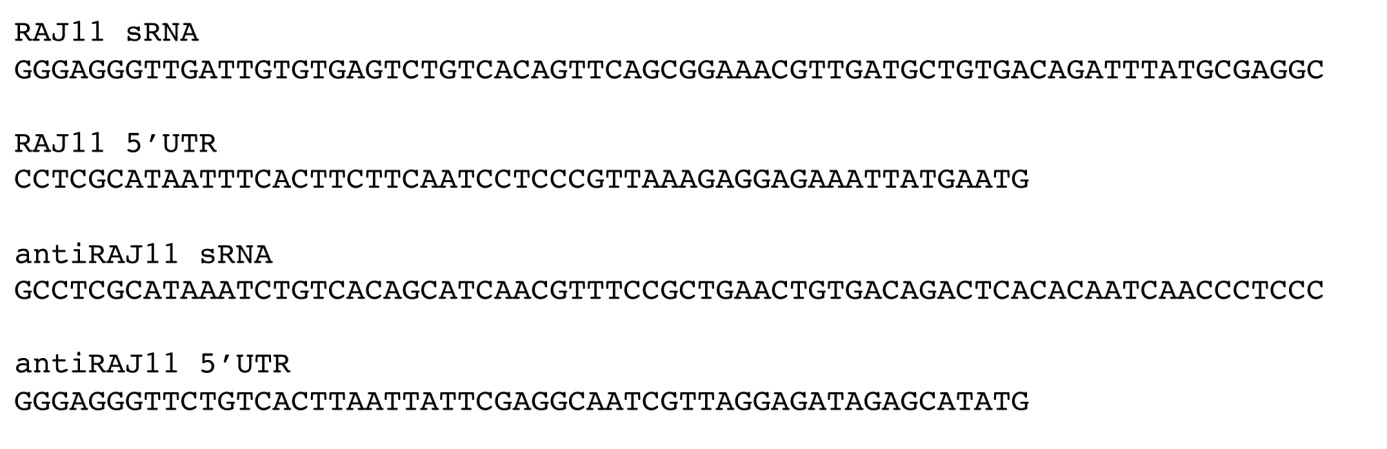


**Figure S2**: Sequences of the RNA species.


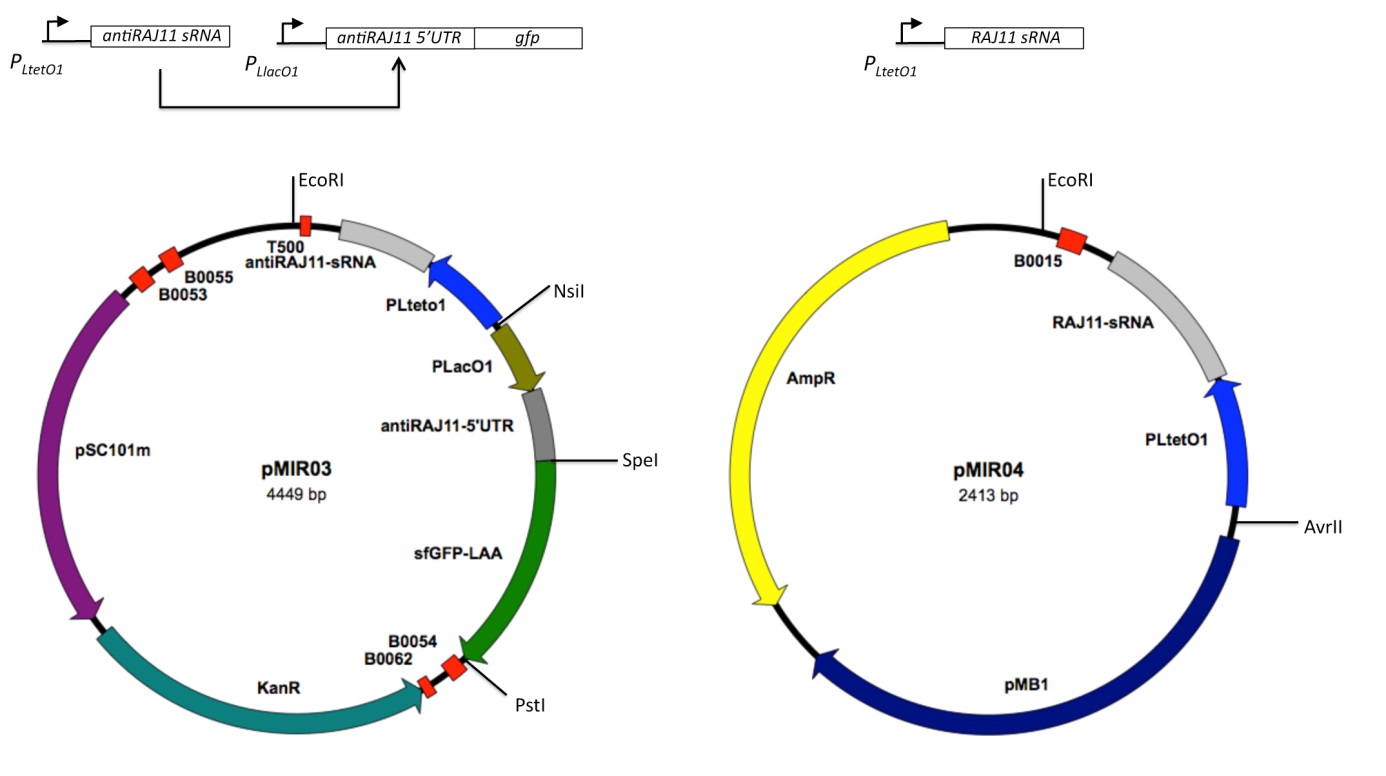


**Figure S3**: Maps of the plasmids used in this work for expressing the designed sRNA

systems.


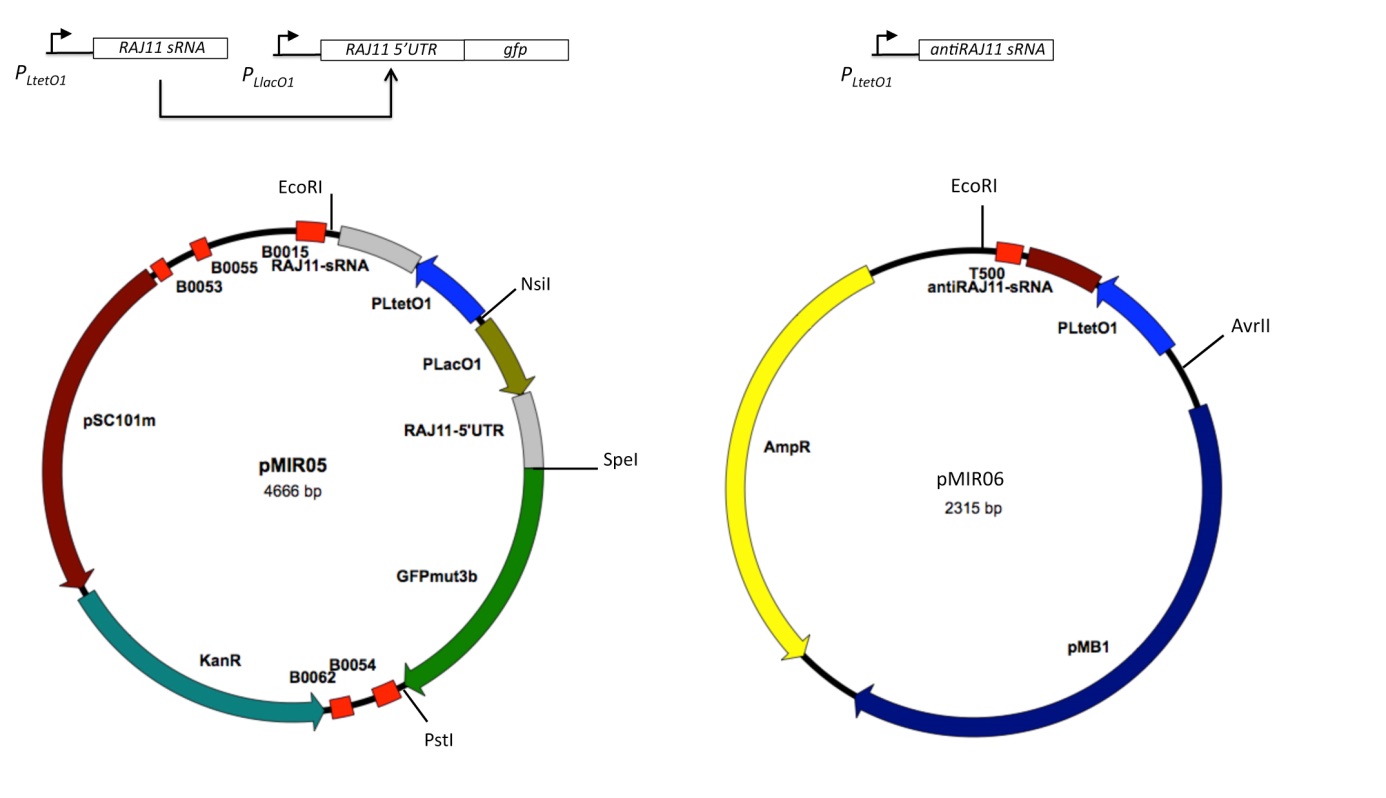


**Figure S4**: Maps of the plasmids used in this work for expressing the designed sRNA

systems.


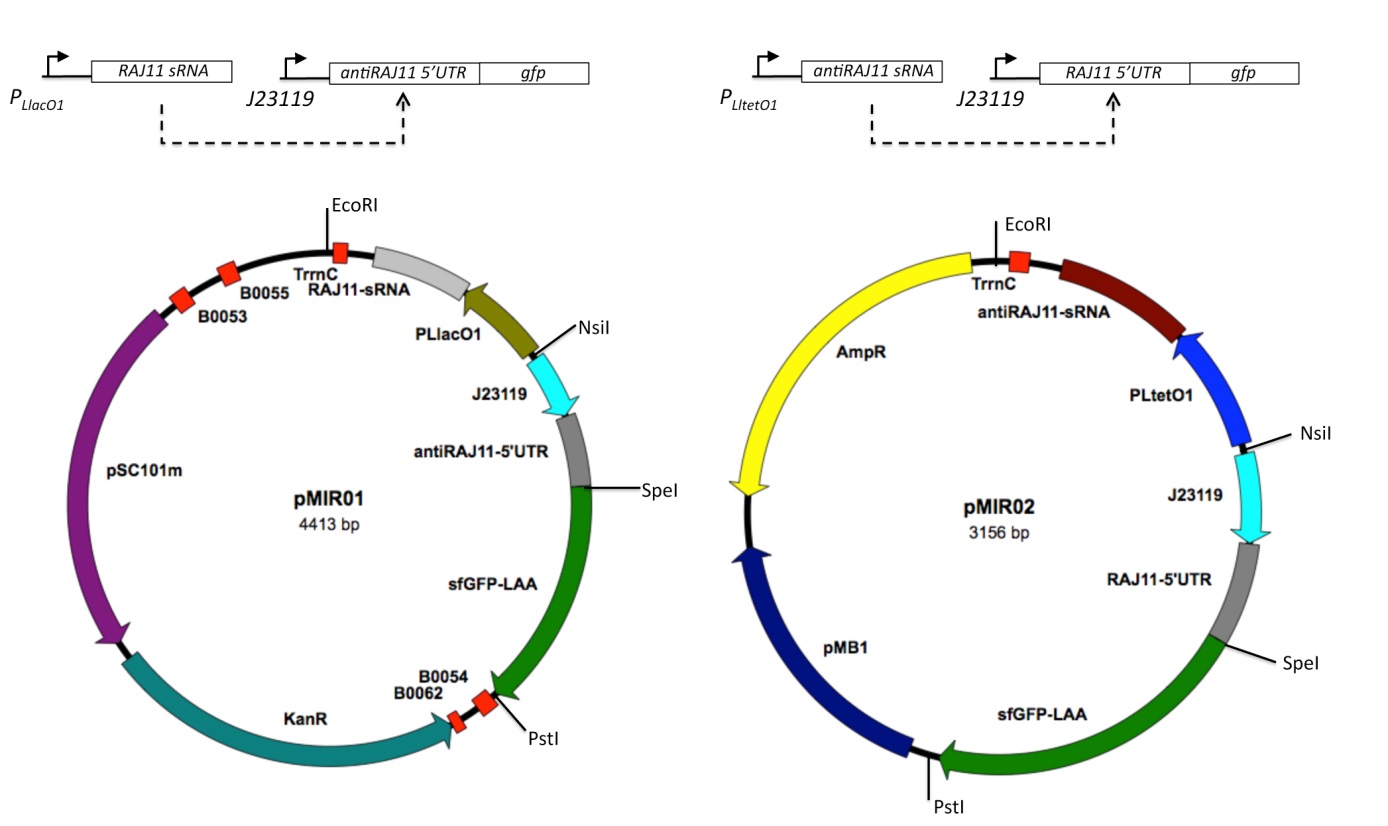


**Figure S5**: Maps of additional plasmids used in this work for studying the orthogonality of the designed sRNA systems.


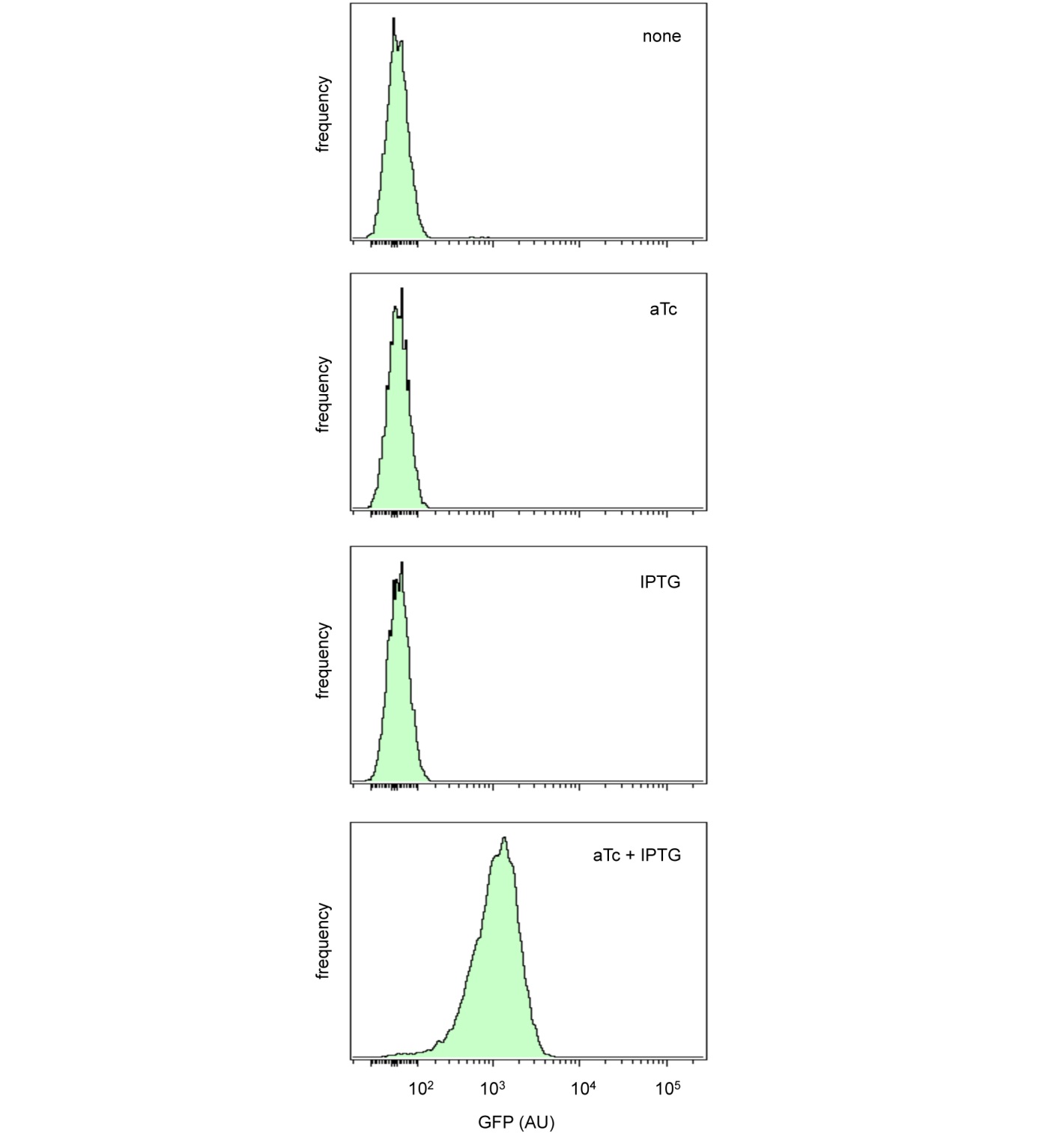


**Figure S6**: Characterization result of antiRAJ11 system by flow cytometry which shows the behavior at the single-cell level.


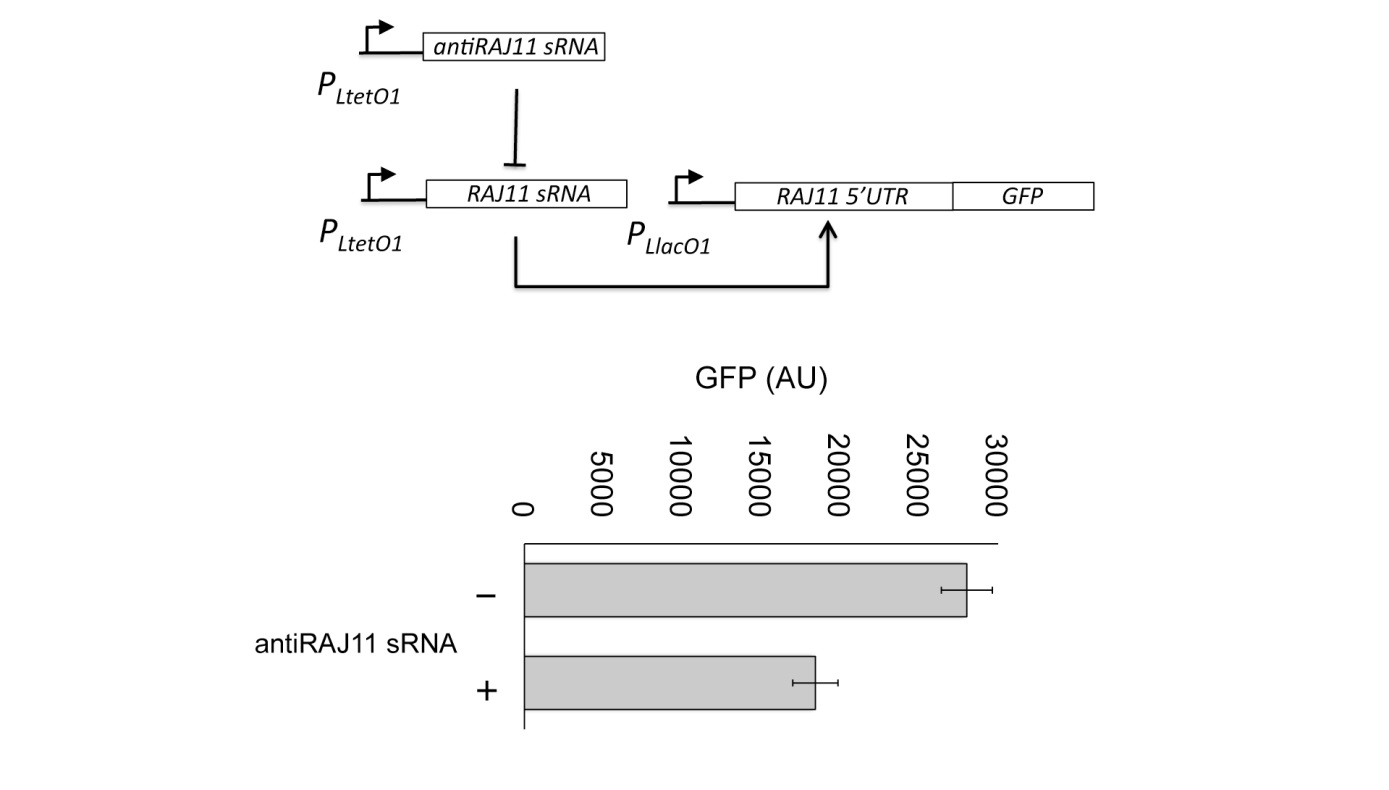


**Figure S7**: Scheme of the regulatory circuit and characterization result of antiRAJ11 sRNA inhibition of the action of RAJ11 sRNA. Here, + (or −) means that the antiRAJ11 sRNA is (or is not) present (introduced with a different plasmid). Assays performed in JS006 cells.


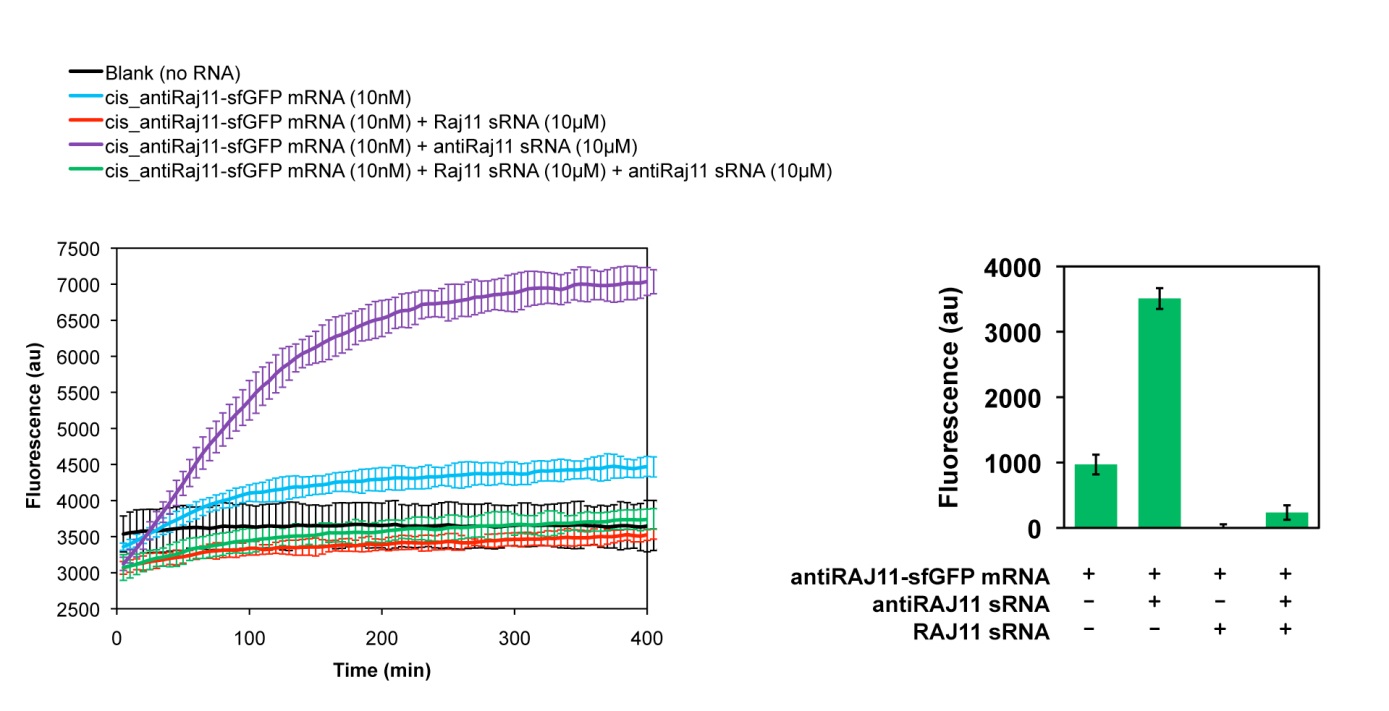


**Figure S8**: On the left, dynamics of in vitro translation (three replicates) for different combinations of RNAs (pre-transcribed). On the right, stationary behavior (background subtraction).

**
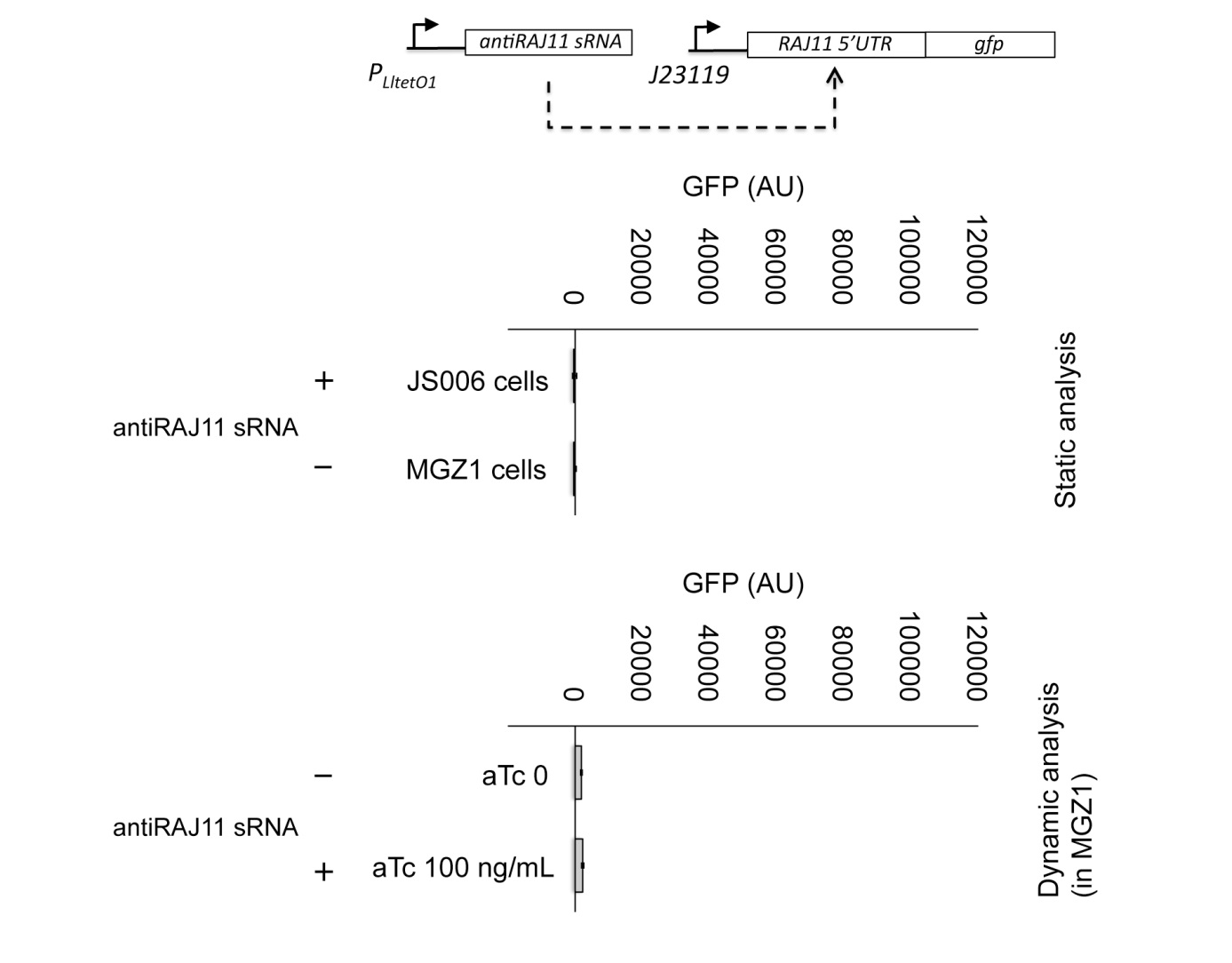
**

**Figure S9**: Scheme of the regulatory circuit and characterization result of antiRAJ11 sRNA transactivation of cis-repressed GFP by RAJ11 5' UTR. + stands for an assay where the sRNA is expressed (performed in JS006 cells or with aTc in MGZ1 cells), while − for an assay where the sRNA is highly repressed (performed in MGZ1 cells).

**
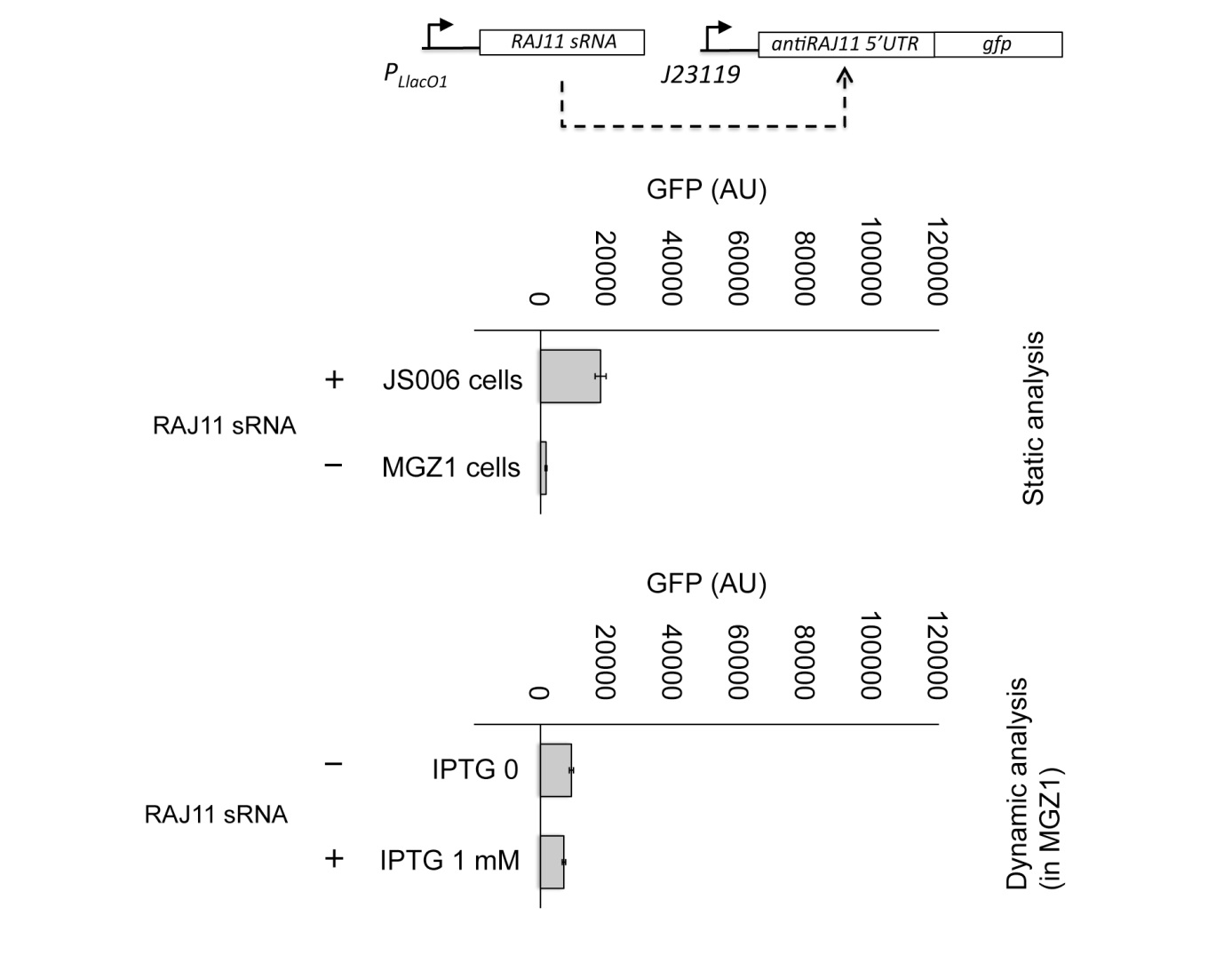
**

**Figure S10**: Scheme of the regulatory circuit and characterization result of RAJ11 sRNA transactivation of cis-repressed GFP by antiRAJ11 5' UTR. + stands for an assay where the sRNA is expressed (performed in JS006 cells or with IPTG in MGZ1 cells), whilst − for an assay where the sRNA is highly repressed (performed in MGZ1 cells).

**Supplementary References**

[1] Rodrigo, G., Landrain, T.E., & Jaramillo, A. De novo automated design of small RNA circuits for engineering synthetic riboregulation in living cells. *Proc. Natl. Acad. Sci. USA* 109, 15271-15276 (2012).

[2] Rostain, W., Landrain, T.E., Rodrigo, G., & Jaramillo, A. Regulatory RNA design through evolutionary computation and strand displacement. *Methods Mol. Biol.* 1244,

63-78 (2015).

[3] Rodrigo, G., & Jaramillo, A. RiboMaker: computational design of conformation-based riboregulation. *Bioinformatics* 30, 2508-2510 (2014).

[4] Kirkpatrick, S., Gelatt, C.D., & Vecchi, M.P. Optimization by simulated annealing.

*Science* 220, 671-680 (1983).

[5] Hofacker, I.L., Fontana, W., Stadler, P.F., Bonhoeffer, L.S., Tacker, M., & Schuster, P.

Fast folding and comparison of RNA secondary structures. *Monatsch. Chem.* 125,

167-188 (1994).

[6] Sambrook, J., Fritsch, E.F. & Maniatis, T. Molecular Cloning: A Laboratory Manual

(Cold Spring Harbor Laboratory Press, New York, 1989).

[7] Shen, S., Rodrigo, G., Prakash, S., Majer, E., Landrain, T.E., Kirov, B., Daròs, J.A., & Jaramillo, A. Dynamic signal processing by ribozyme-mediated RNA circuits to control gene expression. *Nucleic Acids Res*. doi: 10.1093/nar/gkv287 (2015).

[8] Lutz, R., & Bujard, H. Independent and tight regulation of transcriptional units in Escherichia coli via the LacR/O, the TetR/O and AraC/I1-I2 regulatory elements. *Nucleic Acids Res.* 25, 1203-1210 (1997).

[9] Pédelacq, J.-D., Cabantous, S., Tran, T., Terwilliger, T.C., & Waldo, G.S. Engineering and characterization of a superfolder green fluorescent protein. *Nat. Biotechnol.* 24,

79-88 (2003).

[10] Hersch, G.L., Baker, T.A., & Sauer, R.T. SspB delivery of substrates for ClpXP proteolysis probed by the design of improved degradation tags. *Proc. Natl. Acad. Sci. USA* 101, 12136-12141 (2004).

[11] Stricker, J., Cookson, S., Bennett, M., Mather, W., Tsimring, L., & Hasty, J. A fast, robust and tunable synthetic gene oscillator. *Nature* 456, 516-519 (2008).
